# Decoding Visual fMRI Stimuli from Human Brain Based on Graph Convolutional Neural Network

**DOI:** 10.3390/brainsci12101394

**Published:** 2022-10-15

**Authors:** Lu Meng, Kang Ge

**Affiliations:** College of Information Science and Engineering, Northeastern University, Shenyang 110000, China

**Keywords:** brain decoding, convolutional neural network, graph convolution, functional magnetic resonance image

## Abstract

Brain decoding is to predict the external stimulus information from the collected brain response activities, and visual information is one of the most important sources of external stimulus information. Decoding functional magnetic resonance imaging (fMRI) based on visual stimulation is helpful in understanding the working mechanism of the brain visual function regions. Traditional brain decoding algorithms cannot accurately extract stimuli features from fMRI. To address these shortcomings, this paper proposed a brain decoding algorithm based on a graph convolution network (GCN). Firstly, 11 regions of interest (ROI) were selected according to the human brain visual function regions, which can avoid the noise interference of the non-visual regions of the human brain; then, a deep three-dimensional convolution neural network was specially designed to extract the features of these 11 regions; next, the GCN was used to extract the functional correlation features between the different human brain visual regions. Furthermore, to avoid the problem of gradient disappearance when there were too many layers of graph convolutional neural network, the residual connections were adopted in our algorithm, which helped to integrate different levels of features in order to improve the accuracy of the proposed GCN. The proposed algorithm was tested on the public dataset, and the recognition accuracy reached 98.67%. Compared with the other state-of-the-art algorithms, the proposed algorithm performed the best.

## 1. Introduction

As the center of the nervous system, the brain is the most complex and powerful tissue of the human organs [1]; it controls people’s consciousness, processes external stimulation information, and controls advanced activities such as thinking, emotion, and movement [2].

Brain decoding is one of the hotspots in the research of the neuroscience field and involves computational models that describe the correspondence between brain activity patterns and external stimuli and predict external stimulus information from the observed brain response activity [3]. Thus, the main goal of neural decoding is to take a discriminative approach to understanding the measured activity that carries the information produced by different stimuli, and in this study, “activity” refers to the stimulation of human visual areas, as manifested by the formation of functional magnetic resonance imaging.

When humans see different types of images, they produce different stimuli in the visual functional area, and this difference will eventually be reflected in the fMRI images [4]. The purpose of the proposed algorithm is to predict which type of image the subject’s eye is watching by decoding the corresponding subject’s fMRI, which is of great significance for people in understanding the working mechanism of the brain visual function regions. 

With the rapid development of neuroscience and technology, the methods of brain decoding are emerging [5,6,7,8,9,10,11,12]. Many scholars have proposed studies on how the human brain interacts with the outside world. Some of these studies were based on deep learning methods. For example, Hiroshi Ban et al. constructed a deep learning (DL) network that can reconstruct the stimulus image seen by the subject by analyzing the functional magnetic resonance imaging; this reconstructed image had a high similarity to the stimulus image [12]. Horikawa and Kamitani [4] demonstrated that the features obtained by the convolutional neural network (CNN) model at each level were highly correlated with the neural activity of the visual cortex, and they trained a deep CNN model to obtain feature maps of the stimulus image, and then, a regression model was used to match the feature maps with the fMRI to determine what stimulus image the subject saw. Zhao et al. [13] designed a deep 3D-CNN framework for accurately classifying functional brain networks reconstructed from sparse representations of whole-brain fMRI signals. The results of experiments conducted on the Human Connectome Project fMRI dataset demonstrate that this 3D CNN is able to perform functional network classification and recognition tasks effectively. Li et al. proposed C3d-LSTM [14], which combines a series of 3D CNNs to extract the spatial features from 3D fMRI image sequences and then puts the obtained feature maps into a long short-term memory network (LSTM) to decode the time-varying information. Wang et al. [15] constructed graphs based on functional connectivity and presented a connectivity-based graph convolution network (cGCN) architecture for fMRI decoding. Such an approach can accurately extract spatial features from connectomic neighborhoods, whose performance was better than that of the CNN. Xiaoxiao Li et al. [16] proposed a graph neural network framework to analyze functional magnetic resonance images and discover neurological biomarkers. They used two independent fMRI datasets: an Autism Spectrum Disorder (ASD) fMRI dataset and data from the Human Connectome Project (HCP) dataset.

Notably, with regard to the visual brain decoding using fMRI, in 2017 Wen et al. [17] completed the semantic classification of fMRI visual stimulus images by pre-training the AlexNet model to train the mapping of the brain voxel responses to the last layer of features of the network and then passing the predicted voxel features to the softmax classification layer. In 2018, Wen et al. [18] mapped voxel responses to ResNet and to the middle layer features of ResNet by assessing the category selectivity of each voxel in the visual cortex; comparing the cortical representational similarity by assessing the category selectivity of each voxel in the visual cortex; comparing cortical representational similarities and their semantic relationships; and achieving the prediction of fMRI visual information. In 2019, Qiao et al. [19] proposed a method for fMRI data classification and decoding based on the bidirectional recurrent neural network (BRNN), inspired by the bidirectional flow of information processed by the visual system of the human brain, with the forward and backward BRNN modules having bottom-up and top-down characteristics, respectively. The method treats selected voxels in each visual region (V1, V2, V3, V4, and LO) as nodes in the spatial sequence and feeds them into the BRNN module for classification. In 2020, Huang et al. [20] constructed a category decoding model based on the long short-term memory (LSTM) network. The experimental results show that the decoding accuracy of multitemporal visual response signals is significantly higher than that of single-time or short-time visual response signals, and the multitemporal visual response signals induced by natural images contain more semantic category information. 

The above DL algorithms have decoded brain fMRI data and achieved good results. However, there are still some shortcomings: (1) the fMRI has time-serial 4D data, and several volume images are acquired over a period of time, which means that the DL algorithms should focus on both spatial and temporal features of the brain activities that need to be analyzed and decoded. (2) The network layers of the graph convolutional network (GCN) cannot be too deep. The GCN can investigate the topological structure of the brain functional areas and then predict the cognitive state of the brain [21], but the optimal number of layers of the GCN is related to the sparsity of the neighbor matrix; when the number of layers is too deep, over-smoothing will occur [22]. 

Our work is inspired by the cascading processing properties of biological visual pathways. The human brain has evolved and learned over a long period of time to become the most effective biological intelligence system; however, the intelligence of computer vision still needs to be improved. Currently deep networks suffer from low robustness and lack of interpretability and constructing more brain-like deep network models is the key to the current artificial intelligence field. Our study tries to use biological vision mechanisms to build deep network computational models to accomplish a brain visual stimuli decoding task.

The novelties of the proposed method are:

(1) We do not design a graph convolutional neural network for the whole fMRI image but only for those functional areas that are responsive to visual stimuli for processing and analysis, which eliminates the non-visual stimuli areas by using template images and reduces the amount of data processing and avoids the interference of other irrelevant areas to the decoding process.
(2) Through extensive experiments, we find the optimal functional connectivity matrix between each visual functional area of the brain, and this matrix , combined with the multidimensional features of each node obtained from the 3D convolutional neural network by using Formula (1), can well describe the decoding process of visual stimuli to achieve a good classification performance.

## 2. Materials and Methods

**Figure 1:**
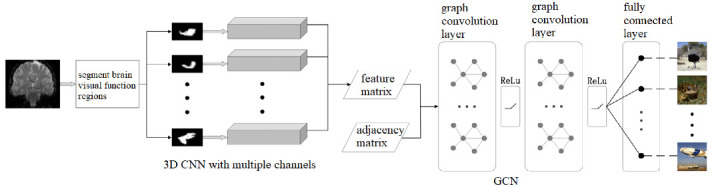
Overall architecture of the proposed algorithm.

The overall architecture of the proposed algorithm is shown in Figure 1. Firstly, we selected eleven ROIs from the human brain visual regions according to the guidance of neurologists (details in Section 2.1). Then, we used a 3D CNN with multiple channels to extract spatial and temporal fMRI features from the eleven ROIs, respectively (details in Section 2.2). Next, we regarded the eleven ROIs as the graph nodes and the functional connections between the ROIs as the graph edges so that we could build a brain network, and we used the GCN to analyze and decode the brain network and predicted what the subjects were watching (details in Section 2.3). In this paper, we use a combination of the 3D convolutional neural network and the graph convolutional neural network to achieve the decoding of functional MR images of visual stimuli, in which we use the 3D CNN to obtain the high-dimensional image features of each visual brain functional area; we use GCN to obtain the relationship between each functional area and combine the results of the 3D CNN and the GCN at the mathematical level through Equation (1). The two networks work together fuse the functional MR image features into the topological information of the visual functional areas to accurately decode the visual information.

### 2.1. Region of Interest

Our work aimed at decoding the fMRI stimulus from vision; therefore, we mainly focused on the visual functional regions of the human brain. Our work was based on the assumption that the human brain visual system is a multi-layer transmission perception system from the concrete to the abstract. The visual system first obtains some concrete features from the external visual stimulus and then obtains more abstract features from these concrete features, by which the human brain can accurately understand the images seen by the subject’s eyes.

**Figure 2:**
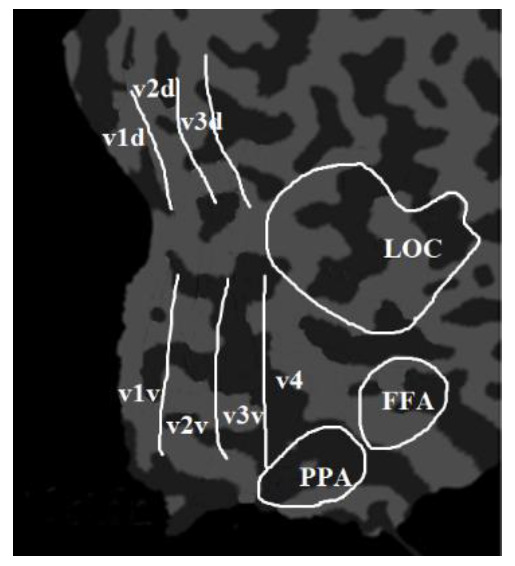
The eleven ROIs of the proposed algorithm, including V1d, V1v, V2d, V2v, V3d, V3v, V4, LOC, FFA, and PPA.

The visual function of the human brain is divided into the primary visual region, the intermediate visual function region, and the advanced visual function region. From primary to advanced, the visual features are more and more abstract and contain more and more semantic image information. Among the visual functional partitions of the human brain, the V1 and V2 regions are mainly responsible for encoding simple image information, such as local orientation and spatial frequency [23]. The V3 region is mainly responsible for the transmission of visual information [24]. The V4 region mainly processes higher-level visual features such as contour, gradient, and semantic information, which plays an important role in object shape information processing, color perception, and object recognition [25]. Furthermore, some studies found that the lateral occipital complex (LOC), the fusiform face area (FFA), and the parahippocampal place area (PPA) had significant responses to advanced visual stimuli such as objects, faces, or scenes [4].To sum up, the main brain response areas to visual stimuli include V1, V2, V3, V4, LOC, FFA, and PPA [1,12,26]. The proposed algorithm took these brain regions as the ROIs and extracted features from these regions. Among them, V1, V2, and V3 were divided into V1d and V1v; V2d and V2v; and V3d and V3v, which were called the low-level visual cortex. The LOC, FFA, and PPA were combined together and named as the higher visual cortex (HVC). Therefore, there are eleven ROIs (V1d, V1v, V2d, V2v, V3d, V3v, V4, LOC, FFA, PPA, and HVC) in the proposed algorithm, which are mainly analyzed and decoded, as shown in Figure 2. 

To accurately segment the eleven ROIs in the fMRI images, we asked neurologists to manually label them and saved the labeling results as template image files. The voxel value of the eleven ROIs in the template image was 1, while the voxel value of other regions was 0.

### 2.2. 3D CNN with Multiple Channels

fMRI data are four-dimensional, and the commonly used the 2D CNN cannot process it. Therefore, this paper used the 3D CNN with multiple channels to extract its features. The resolution of the fMRI dataset used in this algorithm was 64 × 64 × 50 × 3, which meant that the resolution of the two-dimensional slice was 64 × 64, and each volume included 50 slices, and the fMRI scan included 3 volumes. 

**Figure 3:**
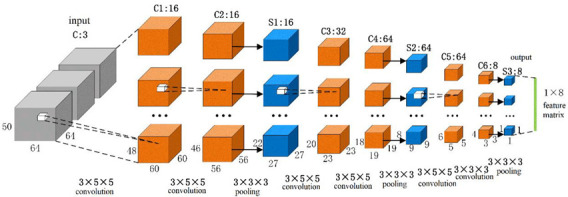
Structure diagram of 3D CNN with multiple channels.

There were eleven ROIs in the proposed algorithm; so, we used eleven 3D CNNs to extract the features of the eleven regions, respectively. The structures of these eleven 3D CNNs were the same, as is shown in Figure 3. The first layer was the input layer, and the size of the input volume of the fMRI data was 64 × 64 × 50, which represented the spatial information, and the number of channels was 3, which represented the temporal information. There were 9 layers in the main network structure, including 6 convolution layers (C1, C2, C3, C4, C5, and C6) and 3 pooling layers (S1, S2, and S3). Each convolution layer was followed by a batch normalization layer (BN) and an activation function layer (ReLU function), and the convolution kernel size was 3 × 5 × 5 (C1, C2, C3, C4, and C5) and 3 × 3 × 3 (C6), and the convolutional step size was 1 × 1 × 1. The pooling layer used the maximum pool, and the size of the pool core was 3 × 3 × 3, and the pooling step size was 2 × 2 × 2. 

The size of each dimension of the feature map obtained after the convolution and pooling operations is calculated as $C=\frac{I+2P-D\times \left(K-1\right)-1}{S}+1$, where *C* denotes the size of each dimension of the output feature map; *I* denotes the size of each dimension of the input data; and *P* denotes the number of filled pixel values at the edges of each dimension of the input. There is no filling in this equation; so, the value of *P* is 0; *D* denotes the spacing between elements in the convolution kernel or pooling kernel (the default is 1); *K* denotes the size of the convolution kernel or pooling kernel; and *S* denotes the step size, and the calculation results are rounded down.

In the 3D CNN, we use a cascade convolution of small convolution kernels followed by a pooling layer for every two convolutional layers because this method can deepen the network depth; cascade convolution can extend the perceptual field, and the feature extraction ability of a cascade convolution of multiple small convolution kernels is the same as that of a single large convolution kernel convolution (the size of the perceptual field is the same). For example, using two 3 × 3 convolutional kernels instead of one 5 × 5 convolutional kernel can reduce the number of parameters and computations, thus increasing the training speed and processing speed of the network. In addition, the cascaded convolution of small convolutional kernels has more nonlinear activation layers, which increases the discriminative power and thus can improve the effectiveness of the network.

The number of channels outputted by each convolution layer was 16, 16, 32, 64, 64, and 8, respectively. The output of one 3D CNN was a 1 × 8 vector, and each ROI was processed by one 3D CNN; therefore, we could obtain the 11 × 8 feature matrix while we combined all the eleven 3D CNN outputs. Next, this 11 × 8 feature matrix was used as one of the inputs to the GCN.

### 2.3. GCN

Based on graph spectrum domain analysis, the proposed GCN’s interlayer propagation rules are shown in Formula (1):

(1) $$H^{\left(l+1\right)}=\sigma \left({\tilde{D}}^{-\frac{1}{2}}\tilde{A}{\tilde{D}}^{-\frac{1}{2}}H^{\left(l\right)}W^{\left(l\right)}\right)$$

where σ() represented the activation function ReLU; $\tilde{A}=A+I_{N}$,$\;\;{\tilde{D}}_{ii}=\underset{j}{\sum }{\tilde{A}}_{ij}$, and *A* represented the adjacency matrix; $I_{N}$ represented the identity matrix; $W^{\left(l\right)}$ represented the parameter matrix that needs to be trained; and $H^{\left(l\right)}$ and $H^{\left(l+1\right)}$ represented the outputs of layer *l* and *l* + 1 of the proposed GCN, respectively.

**Figure 4:**
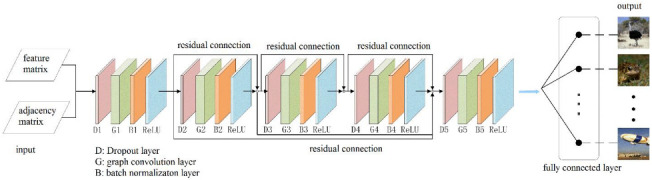
The network structure of the proposed GCN.

The network structure of the proposed GCN is shown in Figure 4, and the inputs to the GCN were one 11 × 8 feature matrix and one 11 × 11 adjacency matrix. The feature matrix was obtained by the 3D CNN with multiple channels in Section 2.2, and the adjacency matrix was defined according to the functional correlation between the eleven ROIs. The whole network was composed of five convolution blocks. Each convolution block successively included a dropout layer (dropout value = 0.5), a graph convolution layer, a BN layer, and an activation function layer (ReLU function). 

The dropout layer can effectively prevent overfitting, and the graph convolution layer makes accurate predictions by matrix multiplication of the input feature matrix, the adjacency matrix, and the parameters for training, so that the feature vectors of each region are linked to each other for fusion. Batch normalization allows the 3D fMRI data distribution to be converted to a normal distribution with mean 0 and variance 1, thus allowing the input of the activation function to fall in the sensitive regions and have the ability to avoid the problem of gradient disappearance. The problem of over-smoothing occurs when the number of layers in a convolutional neural network increases, i.e., the eigenvalues of each node converge to the same value. Therefore, we introduce the residual connection used in the convolutional neural network, which can effectively deepen the depth of the graph convolutional neural network. The final output is classified in the fully connected layer so as to predict the class of the object in the picture seen by the subject based on the fMRI image data.

**Figure 5:**
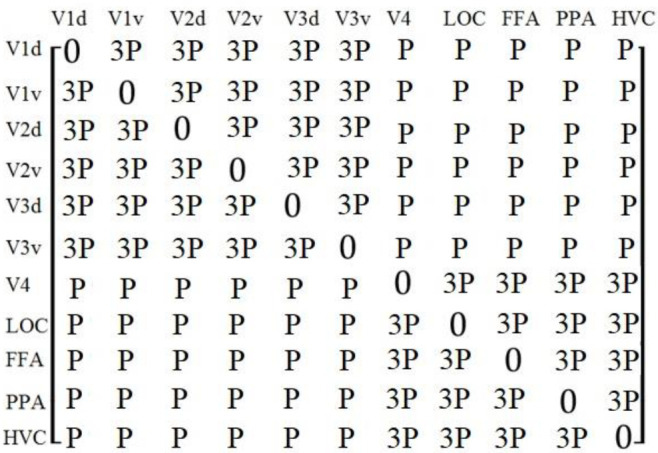
The adjacency matrix in the proposed algorithm, where *P* indicated the Pearson correlation coefficient between ROIs.

The proposed algorithm used the adjacency matrix to reflect the functional correlation between the eleven ROIs. V1, V2, and V3 mainly process the low-level visual features, while V4, LOC, FFA, PPA, and HVC mainly process the medium- and high-level visual features. Therefore, the proposed algorithm divided the eleven ROIs into two groups: V1d, V1v, V2d, V2v, V3d, and V3v, which were called low-level visual regions, and V4, LOC, FFA, PPA, and HVC, which were called medium- and high-level visual regions. We used a Pearson correlation coefficient between ROIs as the functional correlations, and we supposed that the functional correlations from the same group were stronger; therefore, we added larger weights to the coefficients if the ROIs were from the same groups; the adjacency matrix was defined as Formula (2) and is shown in Figure 5.

(2) $$\begin{array}{l} a_{i,j}=\omega \times P(r_{i},r_{j})\\ \omega =\left\{ \begin{array}{l} 3{\;\mathrm{if}\;r}_{i}{\;\mathrm{and}\;r}_{j}\;\mathrm{were}\;\mathrm{in}\;\mathrm{the}\;\mathrm{same}\;\mathrm{groups}\\ 1{\;\mathrm{if}\;r}_{i}{\;\mathrm{and}\;r}_{j}\;\mathrm{were}\;\mathrm{not}\;\mathrm{in}\;\mathrm{the}\;\mathrm{same}\;\mathrm{groups}\\ 0\;\mathrm{if}\;i=j \end{array}\right. \end{array}$$

*P*($r_{i},\;r_{j}$) indicated the Pearson correlation coefficient between $r_{i}$ and $r_{j}$, and ω indicated the weights.

To further verify the validity of the value of *ω* in Formula (2), we made the value of *ω* equal to 1.5, 2.0, 2.5, 3.0, 3.5, 4.0, and tested the accuracy of the brain decoding by the network model in Figure 1, and the results were 81.25%, 84.66%, 93.21%, 98.67%, 97.11%, and 96.89%, respectively. Therefore, we set *ω* = 3. 

We tried two other definitions of the adjacency matrix, such as setting a random 11 × 11 adjacency matrix or calculating the Pearson correlation coefficient between each two ROIs as the elements of the adjacency matrix. The experimental results showed that the prediction accuracy values of these two options were only 35.2% and 46%, respectively, which was far lower than the prediction accuracy of the adjacency matrix defined in Figure 5. We believe that the reasons for the result are that: (1) the random adjacency matrix cannot accurately reflect the correlation between the brain functional regions, which may cause some interference; (2) the Pearson correlation coefficient was used to calculate the linear correlation between the values of the voxels in the ROIs, but the stronger visual stimulus does not necessarily correspond to the higher voxel values.

**Figure 6:**
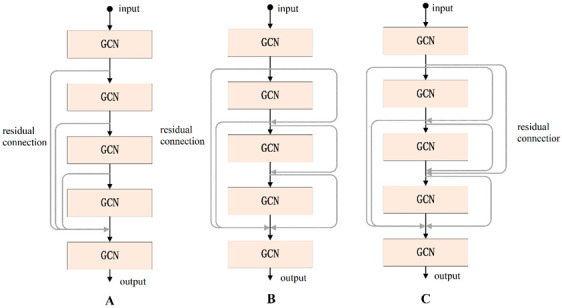
Three types of residual connections.

According to the experimental results, we found that when the number of layers in the GCN increases it will cause gradient disappearance. Therefore, the number of layers in the traditional GCN is generally 2 or 3, and the deeper the number of layers, the worse the performance. To address this problem, we used the residual connection [27] in the proposed GCN, which can effectively deepen the depth of the network. Residual connections have many types of structures, and in this paper, we discuss three types, as shown in Figure 6. Using the same dataset in the same experimental environment, this paper tested these three residual connection methods. The accuracy of type A was 91.55%, that of type B was 98.67%, and that of type C was 93.66%; so, the residual connection mode of type B was finally selected.

### 2.4. Dataset

In the experiment, we used the dataset from reference [4], which can be obtained from https://openneuro.org/datasets/ds001246/versions/1.2.1 (accessed on 10 October 2022). Each scanning session consisted of functional (EPI) and anatomical (inplane T2) data. The functional EPI images covered the entire brain (TR, 3000 ms; TE, 30 ms; flip angle, 80°; voxel size, 3 × 3 × 3 mm; FOV, 192 × 192 mm; number of slices, 50; slice gap, 0 mm) and inplane T2-weighted anatomical images were acquired with the same slices used for the EPI (TR, 7020 ms; TE, 69 ms; flip angle, 160°; voxel size, 0.75 × 0.75 × 3.0 mm; FOV, 192 × 192 mm). The dataset also includes a T1-weighted anatomical reference image for each subject (TR, 2250 ms; TE, 3.06 ms; TI, 900 ms; flip angle, 9°; voxel size, 1.0 × 1.0 × 1.0 mm; FOV, 256 × 256 mm). 

The data were collected by 5 healthy subjects (1 female and 4 males, aged between 23 and 38) with normal vision or vision corrected to normal, and all the subjects had experience and training in the fMRI experiment. Each subject conducted 24 rounds of separate collection; each round lasted for 8 min and 54 s, of which 39 s were in a resting state. There were 55 stimulation blocks in each run, including 50 blocks with different images and 5 randomly interspersed blocks. The whole-brain scan took 3 s at a time, and the duration of each block was 9 s.

**Figure 7:**
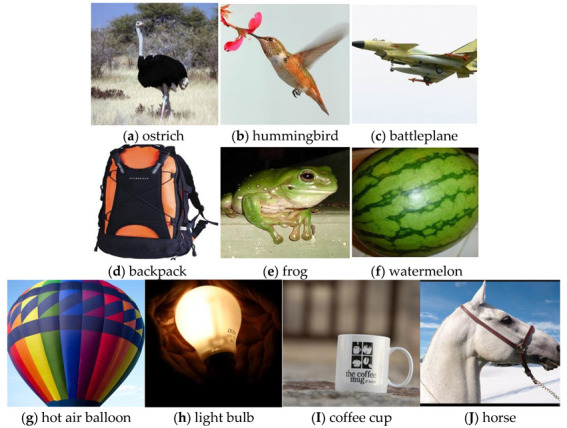
The stimulus images of ten categories from public dataset ImageNet.

The stimulus images used in the experiment were collected from the online image database ImageNet [28]. In this paper, 10 categories of images were used as visual stimulus sources to obtain fMRI images. These ten categories were ostrich, hummingbird, battleplane, backpack, frog, watermelon, hot air balloon, light bulb, coffee cup, and horse, as shown in Figure 7. A total of 522 images from five subjects were collected from the ten categories, as shown in Table 1. There were some differences in the fMRI signals among different subjects which may have influenced the training of the proposed model; therefore, we normalized the image voxel values of the 5 fMRI sequences from different subjects to make the voxel values in the same range. We used different combinations of the training set, validation set, and test set to complete the 5 test trials, and in each trial, we randomly selected images from the three subjects as the training dataset, images from one subject as the validation dataset, and images from one subject as the test dataset. The training parameters of the proposed algorithm were set as shown in Table 2.

## 3. Experimental Results

The software and hardware experimental environment in this paper is shown in Table 3. We programmed the codes in Python3.7, and the neural network was built using PyTorch deep learning framework, which was accelerated by CUDA and cuDNN.

The computing cost of the proposed method is 0.2 s for each fMRI scan. 

### 3.1. Quantitative Evaluation Metric and Network Training 

In the experiments, we used precision, recall, F-score, and accuracy as the quantitative evaluation metrics.

(3) $$precision=\frac{TP}{TP+FP}\;$$

(4) $$recall=\frac{TP}{TP+FN}$$

(5) $$F-Score=\left(1+\beta^{2}\right)\times \frac{precision\times recall}{\left(\beta^{2}\times precision\right)+recall}$$

(6) $$Accuracy=\frac{TP+TN}{TP+FP+TN+FN}$$

where *TP* represented the true positive rate, *FP* represented the false positive rate, *TN* represented the true negative rate, *FN* represented the false negative rate, and *β* = 1.

**Figure 8:**
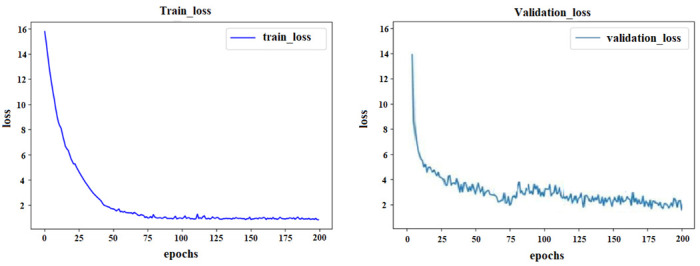
Loss value of the training dataset and validation dataset.

The training parameters of the proposed algorithm were set as shown in Table 2, and the loss value during the training is shown in Figure 8. In the beginning, the loss value decreased rapidly, and with the increase in the number of iterations, the loss value became stable.

### 3.2. Hyperparameters

In this paper, the 3D CNN and the GCN were trained together, and the loss function of the proposed algorithm was the cross-entropy function.

(7) $$H\left(p,q\right)=-\sum_{x}p\left(x\right)\log q\left(x\right)$$

where *p*(*x*) represented the real value, and *Q*(*x*) represented the predicted value; the optimizer used in this paper was the adaptive moment estimation (Adam). Moreover, there were some important parameters to be selected for the proposed algorithm, such as the number of network layers of the 3D CNN, the number of elements of the feature vector obtained by the 3D CNN, and the number of network layers of the GCN.

(1) the number of network layers of the 3D CNN

**Figure 9:**
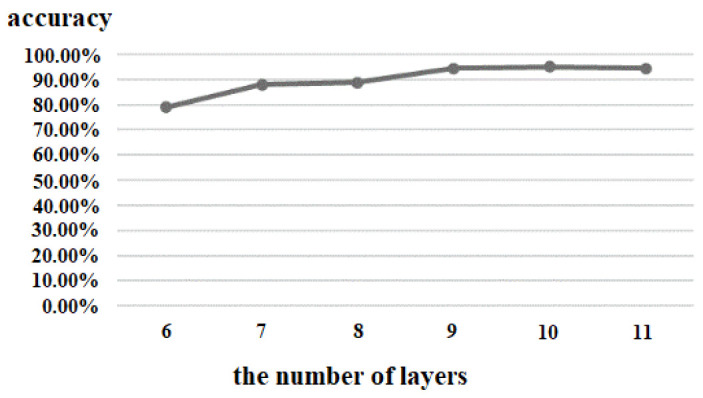
Comparison of different number of layers of 3D CNN.

The number of network layers of the 3D CNN can affect the depth of the network, and we tested six layers (3 convolutions, 3 pooling), seven layers (4 convolutions, 3 pooling), eight layers (5 convolutions, 3 pooling), nine layers (6 convolutions, 3 pooling), ten layers (6 convolutions, 4 pooling), and eleven layers (7 convolutions, 4 pooling) under the same experimental environment. The results are shown in Figure 9. With the increase of the number of layers of the 3D CNN, the accuracy increased gradually, but after reaching 9 layers, the change of accuracy did not grow; that is to say, increasing the number of network layers had little effect on the improvement of the accuracy. At the same time, considering that too many network layers can lead to too many network parameters, higher computational complexity, and lower processing speed, the proposed algorithm therefore set the number of layers to 9.

(2) the number of elements of the feature vector

**Figure 10:**
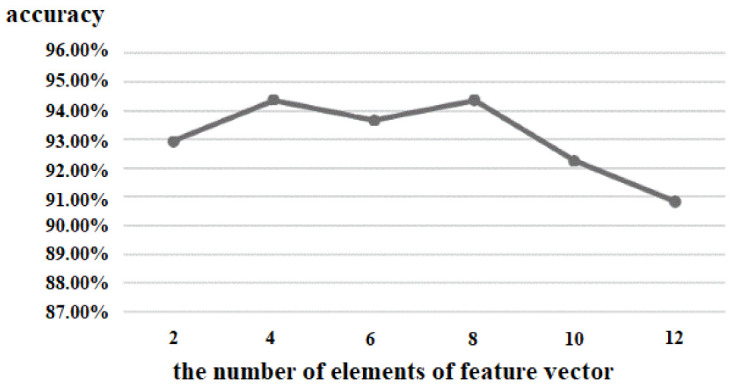
Comparison of the number of elements of the feature vector.

The output of the proposed 3D CNN was a feature matrix, which was composed of eleven feature vectors. Therefore, the number of elements of the feature vector was also an important parameter for the performance of the proposed algorithm. We tested the values from 2 to 12, and the experimental result is shown in Figure 10. When the number of elements of the feature vector was less than four, the accuracy increased with the increase of the number of elements. When the number was between 4 and 8, there was only a small change in accuracy. When the number was between 9 and 12, the accuracy became worse. Finally, we set the number of elements of the feature vector as 8; so, the size of the feature vector was 1 × 8, and the output of the 3D CNN was 11 × 8.

(3) the number of network layers of GCN r

**Figure 11:**
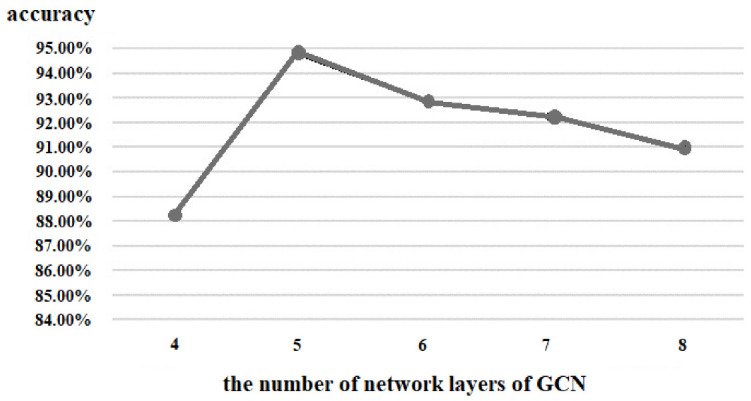
Comparison of the number of network layers of GCN.

Similarly, we also tested the number of layers of the GCN, and the results are shown in Figure 11. The GCN with five layers achieved the highest accuracy; although the accuracy of six layers, seven layers, and eight layers gradually increased, it was still not better than that of five layers. Furthermore, as the layers of the GCN became deeper, the number of parameters also became more and caused more burden for the hardware. Therefore, we set the number of network layers of the GCN as five.

### 3.3. Comparison with Other State-of-the-Art Algorithms

To verify the effectiveness of the proposed algorithm, we tested the trained model on the testing dataset and comprehensively evaluated and analyzed the proposed algorithm by calculating the precision, recall, F-score, and accuracy of each class. At the same time, we compared the proposed algorithm with several other classical deep learning algorithms, including AlexNet-decoding [17], ResNet-decoding [18], BRNN-decoding [19], and LSTM-decoding [20]. All the other networks for comparison were re-trained using the same dataset.

## 4. Discussion

In the present study, we proposed a brain fMRI decoding algorithm based on deep learning methods. With the aim of increasing the accuracy of the decoding results, we extracted the feature matrix using eleven 3D CNNs from the ROIs, delineated the functional connections between the visual brain functional regions, and used residual connections to deepen the proposed GCN. According to the experimental results, the proposed algorithm obtained a good performance.

In the proposed method, we combined the CNN and the GCN, where the role of the CNN was to extract the abstract features of the functional MRI image and describe them in the form of an 8 × 1 feature vector, and the role of the GCN was to analyze the extracted features and brain functional network structure to decode and classify visual fMRI stimuli. Similar combinations of the CNN and the GCN can also be found in the references [29].

Compared with other state-of-the-art algorithms, from Table 4, Table 5, Table 6 and Table 7, we can conclude that the proposed algorithm achieved the best performance in the quantitative evaluation metrics of precision, F-score, and accuracy, and the C3d-LSTM had the best performance in recall. Specifically, in each category, the proposed algorithm performed best in the hummingbird, battleplane, frog, and watermelon, and the C3d-LSTM performed best in hot air balloon, light bulb, coffee cup, and horse. From Table 7, in terms of accuracy, the proposed algorithm, the C3d-LSTM, and the CGCN performed well, and the performances of ResNet and the 3D CNN were not good. Therefore, according to all the evaluation metrics, the proposed algorithm outperformed the other four state-of-the-art algorithms.

There is another class of functional MRI decoding algorithms for graph convolutional neural networks; these are based on node-embedding algorithms [30] that map the high-dimensional nodes of the graph to the low-dimensional space and then perform classifications, such as NetMF, RandNE, Node2Vec, and Walklets. These algorithms construct a graph and consider each subject as a node on the graph and achieve the decoding of functional MRI by classifying each node, which can also achieve good results.

The input channel to the 3D CNN is set to three because each block of the original fMRI data has three fMRI scans, which means that during the fMRI data acquisition each whole-brain scan takes 3 s at a time, and the duration of each block is 9 s. Moreover, to verify whether increasing the number of channels would improve the accuracy of the visual decoding, we added an experiment using an interpolation algorithm between the original 3 channels to obtain 5 and 7 channels of data, respectively, but the experimental results showed that increasing the number of channels did not improve the accuracy of the classification but instead slightly diminished it.

Inspired by references [31,32], the GCN of the algorithm in this paper is constructed by means of the weight matrix in Figure 5, where the individual values give the connection weights between the 11 regions. The reasons for this setting are (1) the degree of correlation between the brain functional areas for visual processing varies, and stronger correlation implies smaller spatial distances or similar functions between the brain functional areas; (2) the processing of visual signals in the human brain is hierarchical, i.e., the v1, v2, v3, and v4 brain functional areas mainly process more elementary visual information, while the LOC, FFA, and PPA brain functional areas, on the other hand, mainly process more advanced visual information, which motivates us to assign stronger weight values to brain functional areas of the same hierarchy. Such a setting brings an increase in visual decoding and classification accuracy. The other two choices are setting the weights randomly or setting the weights according to the Pearson correlation; the experimental results showed that the highest accuracy was achieved by a setting in accordance with Figure 5.

The performance of the 3D CNN algorithm was the worst in the experimental results. The main reason was that the 3D CNN network input was the fMRI image data of the whole brain, while the visual functional regions only covered a small part of the brain. The information of the other non-visual function regions in the fMRI image was not helpful for the final prediction but may have caused interference. The second reason was that the whole network of the 3D CNN was composed of two convolution layers, a pooling layer and a full connection layer. The number of network layers was too few to extract the useful features. ResNet had a deeper network structure, with a total of 18 layers, including 17 convolution layers and a full connection layer, and the features of each layer were fused through residual connections. Therefore, ResNet can extract richer features and achieve better results than the 3D CNN. However, ResNet also inputted fMRI images of the whole brain and did not make use of the temporal information contained in the fMRI data. The C3d-LSTM extracted the spatial features in the fMRI images through a series of 3D CNNs and then inputted them into the LSTM to extract the temporal features. However, the C3d-LSTM did not take advantage of the functional correlations between the ROIs. The proposed algorithm combined all the advantages of the above algorithms. Firstly, eleven brain visual function regions were selected as the ROIs to eliminate the information interference of the other regions, and then, the 3D CNN was used to extract the features of the eleven ROIs. Furthermore, the fMRI images at multiple time points were inputted at the same time to ensure that the temporal features could also be extracted. Finally, the GCN was used to fuse these features based on the functional correlations between the ROIs, and the number of layers of the GCN was increased by using the residual connection. 

All the five algorithms performed best for ostrich, backpack, and horse because these types of images were quite different from the other types and had obvious characteristics. Therefore, when the subjects watched these types of images, the brain’s response to visual stimuli was quite different from that of the other kinds of images. For hummingbird, fighter, watermelon, hot air balloon, and light bulb, the errors of the prediction were relatively high; the main reason was that (1) the shape and appearance of the hummingbird and battleplane were somewhat similar, which may cause a similar response from the brain visual function regions; (2) some watermelons in the stimulus picture were cut off, while some watermelons were not; and (3) the shape of watermelon, hot air balloon, and bulb was oval, which may have led to misprediction among the three types. Therefore, when the shape, color, and background of the object in the stimulus images were quite different, the brain visual stimuli were different when the subjects watched the images, which may have led to the significantly different fMRIs, and the accuracy of the proposed algorithm’s prediction was higher. Conversely, if the subjects were watching two similar objects, the accuracy was reduced [19,33,34].

To illustrate the role of the proposed GCN, we removed the GCN from the proposed algorithm, and the features extracted by eleven 3D CNNs were directly inputted into the fully connected layers for classification without GCN processing, and all the other parameters were kept unchanged. Using the same training and testing dataset, the experimental results illustrated that the use of the GCN effectively improved the accuracy of the network, which increased from 86.62% to 98.67%. The main reasons were as follows: (1) the GCN multiplied the feature matrix (obtained from the 3D CNN) with the adjacency matrix (defined by visual functional connections) and added appropriate weights (the weights were automatically learned after training) to make this feature information fuse well and make full use of the characteristics of the functional correlations between the ROIs; (2) the GCN can increase the depth of the whole network so that the network can extract richer and deeper features.

To illustrate the role of the residual connections in the proposed GCN, we built another graph convolution network without residual connections, which was named the GCN-noRC. We compared the proposed GCN with the GCN-noRC using the same experimental environment and the same dataset and found that the accuracy of the proposed GCN was 98.67% and that of the GCN-noRC was 85.21%, which indicated that residual connections can alleviate the phenomenon of gradient disappearance and effectively improve the accuracy of the graph convolution network.

The limitations of the proposed algorithm are mainly reflected in the following three aspects: (1) the sample size is limited, and the same patients belong to the training and test set, which may weaken the generalizability; in the next step, we will collect more data from more subjects. (2) The number of classification types for brain decoding is only ten, and we will enlarge it in the future. (3) The lengths of the dataset are only three for each fMRI block, which restricts the accuracy of the classification, and the proposed algorithm cannot be compared with other datasets with different block lengths.

Regarding future work, we will make the proposed algorithm have better transferability, i.e., the data collected between different groups of subjects, and among different sites using distinct scanning sequences, can be applied to the model proposed in this paper, which is inspired by reference [35] that uses transfer learning to build GCNs and investigate the transferability of deep artificial neural networks in brain decoding.

## 5. Conclusions

Aiming at the challenges and shortcomings of brain decoding algorithms, we proposed a brain decoding algorithm of fMRI based on a graph convolution neural network. The main innovations of the proposed algorithm were (1) the extraction of the image features of fMRI using the 3D CNN; (2) the construction of the GCN according to the functional connections between the visual functional regions of the human brain; (3) the combination of the CNN and the GCN to deepen the network. The experimental results showed that the prediction accuracy of the proposed algorithm was 98.67% and outperformed the other state-of-the-art algorithms.

## Figures and Tables

**Table 1:** The number of images in each category.

Item	Number of Images	Label
ostrich	57	1
frog	51	2
hummingbird	48	3
backpack	54	4
horse	52	5
coffee cup	57	6
battleplane	47	7
hot air balloon	48	8
light bulb	56	9
watermelon	52	10
total	522	

**Table 2:** The training parameters of the proposed algorithm.

Training Parameters	Setting Values
Epoch	200
Learn rate	0.01
Weight decay	0.001
Batch size	16
Dropout rate	0.5
Optimizer	Adam

**Table 3:** Experimental environment of software and hardware.

Item	Configuration
Operation system	Ubuntu 16.04
CPU	Intel(R) Xeon(R) E5-1650 v3 @ 3.50GHz
Memory	128G
GPU	TITAN RTX
Video memory	24G
Hard disk	4TB
Software	Python3.7; Pytorch1.4.0; CUDA 10.0.130; CUDNN 765
Compiler	Pycharm

**Table 4:** Comparison of experimental results (precision, %).

Methods	Average Performance	Ostrich	Hummingbird	Battleplane	Backpack	Frog	Watermelon	Hot Air Balloon	Light Bulb	Coffee Cup	Horse
AlexNet-decoding [17]	82.84	95.51	72.49	100	94.25	61.15	67.21	46.07	67.03	93.32	93.31
ResNet-decoding [18]	93.50	100	85.79	89.21	100	100	84.57	88.25	83.83	100	100
BRNN-decoding [19]	86.72	100	79	78.27	100	93.19	75.94	60.74	89.89	100	88.45
LSTM-decoding [20]	92.05	100	92.31	90.15	100	87.41	84.92	78.53	80.95	100	100
CNN-decoding [4]	88.57	100	70	83.33	72.72	72.72	77.78	84.61	90.91	63.63	100
Ours	93.33	100	90	100	100	100	83.30	73.30	100	100	100

**Table 5:** Comparison of experimental results (recall, %).

Methods	Average Performance	Ostrich	Hummingbird	Battleplane	Backpack	Frog	Watermelon	Hot Air Balloon	Light Bulb	Coffee Cup	Horse
AlexNet-decoding [17]	75.98	95.06	100	50.07	94.40	100	52.77	56.58	40.22	77.85	93.28
ResNet-decoding [18]	93.12	100	93.03	80.92	100	93.58	84.36	78.32	100	100	100
BRNN-decoding [19]	86.34	100	85.12	70.41	100	85.91	79.51	67.44	80.19	89.00	100
LSTM-decoding [20]	91.23	100	92.51	90.38	100	93.53	84.20	77.96	80.54	95.03	100
CNN-decoding [4]	90.27	89.78	97.78	83.33	80	100	83.63	84.61	83.33	77.78	81.82
Ours	93.33	100	100	91.7	100	100	90.9	84.60	75	100	100

**Table 6:** Comparison of experimental results (F-Score, %).

Methods	Average Performance	Ostrich	Hummingbird	Battleplane	Backpack	Frog	Watermelon	Hot Air Balloon	Light Bulb	Coffee Cup	Horse
AlexNet-decoding [17]	77.75	75.66	45.73	30.37	63.87	55.23	50.52	37.25	57.2	66.60	95.93
ResNet-decoding [18]	93.64	100	89.48	84.85	100	96.32	84.45	83.10	91.14	100	100
BRNN-decoding [19]	86.12	100	81.98	73.78	100	89.26	77.48	63.63	84.38	94.50	93.76
LSTM-decoding [20]	91.37	100	92.67	90.31	100	90.38	84.45	77.99	80.35	97.94	100
CNN-decoding [4]	89.48	90	83.33	90	76.19	86.67	83.33	90.91	73.68	90	70
Ours	93.30	100	94.70	95.70	100	100	86.90	78.60	85.70	100	100

**Table 7:** Comparison of experimental results (accuracy, %).

Methods	Total Accuracy
AlexNet-decoding [17]	82.37%
ResNet-decoding [18]	96.60%
BRNN-decoding [19]	97.10%
LSTM-decoding [20]	93.50%
CNN-decoding [4]	91.25%
Ours	98.67%

## Data Availability

Not applicable.

## References

[1] Rosenke M., van Hoof R., Hurk J.V.D., Grill-Spector K., Goebel R. (2021). A probabilistic functional atlas of human occipito-temporal visual cortex. Cereb. Cortex.

[2] Zatorre R.J., Fields R.D., Johansen-Berg H. (2012). Plasticity in gray and white: Neuroimaging changes in brain structure during learning. Nat. Neurosci..

[3] van Gerven M.A., Kok P., de Lange F.P., Heskes T. (2011). Dynamic decoding of ongoing perception. Neuroimage.

[4] Horikawa T., Kamitani Y. (2017). Generic decoding of seen and imagined objects using hierarchical visual features. Nat. Commun..

[5] Friston K.J., Holmes A.P., Poline J.B. (1995). Analysis of fMRI Time-Series Revisited. Neuroimage.

[6] Richiardi J., Eryilmaz H., Schwartz S. (2011). Decoding brain states from fMRI connectivity graphs. NeuroImage.

[7] Kamitani Y., Tong F. (2005). Decoding the visual and subjective contents of the human brain. Nat. Neurosci..

[8] Haxby J.V., Gobbini M.I., Furey M.L. (2001). Distributed and overlapping representations of faces and objects in ventral temporal cortex. Science.

[9] Tahmassebi A., Gandomi A.H., Schulte M.H. (2018). Optimized naive-bayes and decision tree approaches for fmri smoking cessation classification. Complexity.

[10] Haynes J.D., Rees G. (2005). Predicting the orientation of invisible stimuli from activity in human primary visual cortex. Nat. Neurosci..

[11] Wang M.Y., Li C.L., Zhang W.J. (2019). Support vector machine for analyzing contributions of brain regions during task-state fMRI. Front. Neuroinform..

[12] Ban H., Yamamoto H., Hanakawa T., Urayama S., Aso T., Fukuyama H., Ejima Y. (2013). Topographic Representation of an Occluded Object and the Effects of Spatiotemporal Context in Human Early Visual Areas. J. Neurosci..

[13] Zhao Y., Dong Q., Zhang S. (2017). Automatic recognition of fmri-derived functional networks using 3-d convolutional neural networks. IEEE Trans. Biomed. Eng..

[14] Li W., Lin X.F., Chen X. (2020). Detecting Alzheimer’s disease Based on 4D fMRI: An exploration under deep learning framework. Neurocomputing.

[15] Wang L.B., Li K.M., Hu X.P.P. (2021). Graph convolutional network for fMRI analysis based on connectivity neighborhood. Netw. Neurosci..

[16] Li Y.Z., Dvornek N., Zhang M., Gao S., Zhuang J., Scheinost D., Staib L., Ventola P., Duncan J. (2020). BrainGNN: Interpretable Brain Graph Neural Network for fMRI Analysis. BioRxiv.

[17] Wen H., Shi J., Zhang Y., Lu K.H., Cao J., Liu Z. (2018). Neural Encoding and Decoding with Deep Learning for Dynamic Natural Vision. Cereb. Cortex.

[18] Wen H., Shi J., Chen W., Liu Z. (2018). Deep Residual Network Predicts Cortical Representation and Organization of Visual Features for Rapid Categorization. Sci. Rep..

[19] Qiao K., Chen J., Wang L., Zhang C., Zeng L., Tong L., Yan B. (2019). Category Decoding of Visual Stimuli From Human Brain Activity Using a Bidirectional Recurrent Neural Network to Simulate Bidirectional Information Flows in Human Visual Cortices. Front. Neurosci..

[20] Huang W., Yan H., Wang C., Li J., Yang X., Li L., Zuo Z., Zhang J., Chen H. (2020). Long short-term memory-based neural decoding of object categories evoked by natural images. Hum. Brain Mapp..

[21] Du B., Cheng X., Duan Y., Ning H. (2022). fMRI Brain Decoding and Its Applications in Brain–Computer Interface: A Survey. Brain Sci..

[22] Klicpera J., Bojchevski A., Günnemann S. (2019). Predict then Propagate: Graph Neural Networks meet Personalized PageRank. arXiv.

[23] Kay K.N., Naselaris T., Prenger R.J. (2008). Identifying natural images from human brain activity. Nat. Commun..

[24] Nakamura K., Colby C.L. (2000). Visual, saccade-related, and cognitive activation of single neurons in monkey extrastriate area V3A. J. Neurophysiol..

[25] Tootell R.B., Hadjikhani N. (2001). Where is ‘dorsal V4’in human visual cortex?. Retinotopic, topographic and functional evidence. Cereb. Cortex.

[26] Mantini D., Hasson U., Betti V., Perrucci M.G., Romani G.L., Corbetta M., Orban G.A., Vanduffel W. (2012). Interspecies activity correlations reveal functional correspondence between monkey and human brain areas. Nat. Methods.

[27] He K.M., Zhang X.Y., Ren S.Q., Sun J. Deep residual learning for image recognition. Proceedings of the IEEE Conference on Computer Vision and Pattern Recognition (CVPR).

[28] Deng J., Dong W., Socher R. Imagenet: A large-scale hierarchical image database. Proceedings of the IEEE Conference on Computer Vision and Pattern Recognition.

[29] Feng N.A., Hu F., Wang H., Zhou B. (2021). Motor Intention Decoding from the Upper Limb by Graph Convolutional Network Based on Functional Connectivity. Int. J. Neural Syst..

[30] Saeidi M., Karwowski W., Farahani F.V., Fiok K., Hancock P.A., Sawyer B.D., Christov-Moore L., Douglas P.K. (2022). Decoding Task-Based fMRI Data with Graph Neural Networks, Considering Individual Differences. Brain Sci..

[31] Papadimitriou A., Passalis N., Tefas A. (2019). Visual representation decoding from human brain activity using machine learning: A baseline study. Pattern Recognit. Lett..

[32] TMullen K. (2019). The response to colour in the human visual cortex: The fMRI approach. Curr. Opin. Behav. Sci..

[33] Park S., Brady T.F., Greene M.R., Oliva A. (2011). Disentangling Scene Content from Spatial Boundary: Complementary Roles for the Parahippocampal Place Area and Lateral Occipital Complex in Representing Real-World Scenes. J. Neurosci..

[34] Steel A., Billings M.M., Silson E.H., Robertson C.E. (2021). A network linking scene perception and spatial memory systems in posterior cerebral cortex. Nat. Commun..

[35] Zhang Y., Bellec P. (2020). Transferability of Brain decoding using Graph Convolutional Networks. BioRxiv.

